# Supplementary material for: Follicular Lymphoma Microenvironment Traits Associated with Event-Free Survival
Source: Int J Mol Sci. 2023 Jun 8;24(12):9909. doi: 10.3390/ijms24129909 (PMC10297948; doi:10.3390/ijms24129909)
Supplement: Supplementary file 1 [file ijms-24-09909-s001.zip › ijms-2311066-supplementary-english.pdf]

Supplementary Materials and Methods

Gene expression profiling

RNA was isolated from 18 FFPE tumor samples with the AllPrep DNA/RNA FFPE kit (Qiagen, Hilden, Germany) and quantified by Nanodrop. The RNA quality was checked before performing the Pancancer IO 360 panel (NanoString Technologies, Seattle, WA, USA), and used according to the manufacturer's instructions to analyze the expression of 770 genes and the tumor inflammation signature (TIS) [1]. The gene expression profile of samples was performed with 300 ng of RNA input on the NanoString platform (NanoString Technologies), using the "high sensitivity" setting on the nCounter™PrepStation and 550 field of view (FOV) on the nCounter™Analyzer. Gene expression data were normalized using a ratio of the expression value to the geometric mean of all housekeeping genes on the panel. Housekeeper-normalized data were then log2 transformed. To identify genes differentially expressed between categories, a moderated t-test was applied. P-values were adjusted using the Benjamini and Yekutieli false discovery rate (FDR) method.

Bisulfite conversion and DNA methylation assay

Genomic DNA (900 ng) was bisulfite converted by using the EZ DNA Methylation Kit (D5001—Zymo Research Corporation) according to the manufacturer's protocol. Illumina-specific incubation conditions were applied to improve conversion efficiency. Evaluation of conversion yield was performed by measuring bisulfite-converted DNA (bsDNA) through a single-strand quantification using an N60 Implen Nanophotometer. 200 ng/ul of bisulfite-converted DNA were used for hybridization on Illumina Infinium Methylation EPIC BeadChip. Samples were processed according to the manufacturer's protocols in a semi-automated procedure. Chips were scanned using the Illumina iScan scanner: the fluorescence intensities were stored as intensity data files (*.idat). The methylation score for each CpG site is represented as β values according to the fluorescent intensity ratio between methylated and unmethylated probes. β values may range between 0 (non-methylated) and 1 (fully methylated).

Differential methylation analyses

Group level comparison was conducted by using the RnBeads package in the R environment [2]. Quality control, normalization, and exploratory (e.g., Principal Component Analysis) analyses of raw data were carried out as the first step. After the exclusion of some samples, the final population (controls and cases) was constituted of 25 methylation profiles. Filtering steps removed SNP-enriched (n=139721) and cross-reactive probes (n=34264) and unreliable measurements (n=66272 sites). Moreover, context-specific (n=843), sex chromosomes (n=14082), and probes with many missing values (n=136) were also filtered out. As a final outcome of the filtering procedures, 611577 probes were retained. Signal intensities were normalized using the BMIQ (Beta MIxture Quantile) normalization method [3]. Differential methylation analysis was performed according to the variable sample group by computing p-values using the limma method for the site level analysis. For the analysis of predefined (genes, promoters, CpG island, tiling) regions, a combined p-value was calculated from the p-values of single sites. To avoid potential confounding factors sex variable was used as a covariate in the differential methylation module. Adopting a complementary strategy, stochastic epigenetic mutation (SEM) analysis was also used to estimate epigenetic drift at a single sample level. This analysis approach, developed by Gentilini et al., is widely used in several studies [4–8] and is applied to single (individual) methylation profiles to identify, through a non-parametric statistical approach, single aberrant methylation values (extreme outliers—SEMs) according to a reference methylation range (obtained from the entire lymphoma population). Thresholds are calculated as follows: upper value=Q3+(k*IQR); lower value=Q1-(k*IQR); where Q1=first quartile, Q3=third quartile, IQR (Interquartile range)=Q3-Q1 and k=3. For each sample, extreme outlier values of single methylation profiles were annotated and classified as hyper-methylated or hypo-methylated to controls' relative probe median values. The burden of SEMs was evaluated between the two groups to identify potential differences in epigenetic drift. To detect SEM-enriched regions, for each sample, an over-representation analysis was conducted as described in [Guida et al.]. Results were annotated by using wANNOVAR tool. Computational tool Phenolyzer (phenotype-based gene analyzer) (http://phenolyzer.wglab.org/) [9] prioritized univocal gene

lists by selecting appropriate disease terms (e.g., follicular_lymphoma, immune_system (HPO), tumor (HPO)).

AEC stain validation across rounds

AEC stains for CD8, CD4, PD1, MS4A4A, CD68, CD163, and CD56 were validated on tissue microarrays (TMAs) containing different positive control tissues or on human tonsil (Figure S6a). Each marker was approved by an expert Pathologist (F.L.) upon comparison with the DAB staining. Detection of positive cells for each round was performed using QuPath's Simple Tissue Detection and Positive Cell Detection methods as previously reported [10].

Statistical Analysis

The Generalized Linear Regression (glm) model was adopted to evaluate differences between sample groups. Data were log10 transformed to address skewed/non-normal data. Alternatively, the non-parametric Wilcoxon-Mann–Whitney test was used. Unless otherwise stated, the statistical significance threshold was set to 0.05.

References

1. Ayers, M.; Lunceford, J.; Nebozhyn, M.; Murphy, E.; Loboda, A.; Kaufman, D.R.; Albright, A.; Cheng, J.D.; Kang, S.P.; Shankaran, V.; et al. IFN-γ-Related MRNA Profile Predicts Clinical Response to PD-1 Blockade. *J. Clin. Invest.* **2017**, *127*, 2930–2940, doi:10.1172/JCI91190.
2. Müller, F.; Scherer, M.; Assenov, Y.; Lutsik, P.; Walter, J.; Lengauer, T.; Bock, C. RnBeads 2.0: Comprehensive Analysis of DNA Methylation Data. *Genome Biol.* **2019**, *20*, 1–12, doi:10.1186/s13059-019-1664-9.
3. Teschendorff, A.E.; Marabita, F.; Lechner, M.; Bartlett, T.; Tegner, J.; Gomez-Cabrero, D.; Beck, S. A Beta-Mixture Quantile Normalization Method for Correcting Probe Design Bias in Illumina Infinium 450 k DNA Methylation Data. *Bioinformatics* **2013**, *29*, 189–196, doi:10.1093/bioinformatics/bts680.
4. Gentilini, D.; Somigliana, E.; Pagliardini, L.; Rabellotti, E.; Garagnani, P.; Bernardinelli, L.; Papaleo, E.; Candiani, M.; Di Blasio, A.M.; Viganò, P. Multifactorial Analysis of the Stochastic Epigenetic Variability in Cord Blood Confirmed an Impact of Common Behavioral and Environmental Factors but Not of in Vitro Conception. *Clin. Epigenetics* **2018**, *10*, 1–13, doi:10.1186/s13148-018-0510-3.
5. Gentilini, D.; Garagnani, P.; Pisoni, S.; Bacalini, M.G.; Calzari, L.; Mari, D.; Vitale, G.; Franceschi, C.; Di Blasio, A.M. Stochastic Epigenetic Mutations (DNA Methylation) Increase Exponentially in Human Aging and Correlate with X Chromosome Inactivation Skewing in Females. *Aging (Albany. NY).* **2015**, *7*, 568–578, doi:10.18632/aging.100792.
6. Gentilini, D.; Scala, S.; Gaudenzi, G.; Garagnani, P.; Capri, M.; Cescon, M.; Grazi, G.L.; Bacalini, M.G.; Pisoni, S.; Dicitore, A.; et al. Epigenome-Wide Association Study in Hepatocellular Carcinoma: Identification of Stochastic Epigenetic Mutations through an Innovative Statistical Approach. *Oncotarget* **2017**, *8*, 41890–41902, doi:10.18632/oncotarget.17462.
7. Guida, V.; Calzari, L.; Fadda, M.T.; Piceci-Sparascio, F.; Digilio, M.C.; Bernardini, L.; Brancati, F.; Mattina, T.; Melis, D.; Forzano, F.; et al. Genome-Wide DNA Methylation Analysis of a Cohort of 41 Patients Affected by Oculo-Auriculo-Vertebral Spectrum (OAVS). *Int. J. Mol. Sci.* **2021**, *22*, 1–19, doi:10.3390/ijms22031190.

8. Spada, E.; Calzari, L.; Corsaro, L.; Fazia, T.; Mencarelli, M.; Di Blasio, A.M.; Bernardinelli, L.; Zangheri, G.; Vignali, M.; Gentilini, D. Epigenome Wide Association and Stochastic Epigenetic Mutation Analysis on Cord Blood of Preterm Birth. *Int. J. Mol. Sci.* **2020**, *21*, 1–14, doi:10.3390/ijms21145044.
9. Yang ,Hui.; Robinson, P.N.; Wang, K. Phenolyzer: Phenotype-Based Prioritization of Candidate Genes for Human Diseases. *Nat Methods.* **2015**,*12*, 841–843, doi:10.1038/nmeth.3484.
10. Bulgarelli, J.; Tazzari, M.; Granato, A.M.; Ridolfi, L.; Maiocchi, S.; de Rosa, F.; Petrini, M.; Pancisi, E.; Gentili, G.; Vergani, B.; et al. Dendritic Cell Vaccination in Metastatic Melanoma Turns "Non-T Cell Inflamed" Into "T-Cell Inflamed" Tumors. Front. Immunol. 2019, 10, doi:10.3389/fimmu.2019.02353.
