# Supplementary material for: Follicular Lymphoma Microenvironment Traits Associated with Event-Free Survival
Source: Int J Mol Sci. 2023 Jun 8;24(12):9909. doi: 10.3390/ijms24129909 (PMC10297948; doi:10.3390/ijms24129909)
Supplement: Supplementary file 1 [file ijms-24-09909-s001.zip › Supplementary Figures_IJMS_REVISED2.pdf]

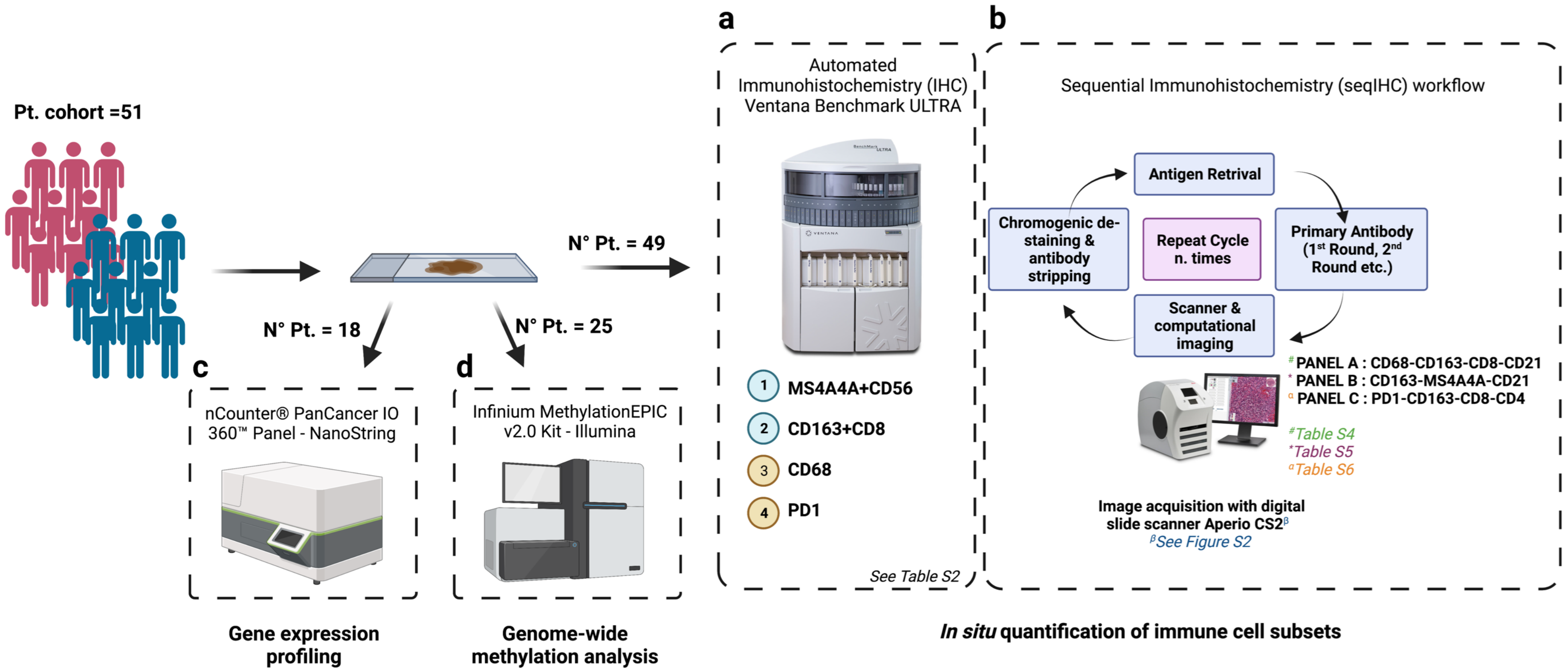

Figure S1. Summary of the techniques and patient population. 49 FFPE blocks obtained at the time of diagnosis were enrolled for the retrospective IHC investigation, 18 for the gene expression analysis and 25 for the methylation profiling after 51 patients passed the inclusion screening. **(a)** Using the Ventana Benchmark ULTRA staining equipment, automated IHC was carried out as follows: 1, 2: double staining; 3, 4 single staining. **(b)** Sequential IHC workflow was conducted manually following iterative cycles of: antigen retrival, antibody labeling, whole slide image scanning and stripping for each antibody. Complete staining removal is confirmed by incubating slides with only the detection reagent. The ideal marker (Round) order for the panel is then established. Refer to Figure S2 for information on computational image processing analysis and pseudo-color picture visualization. **(c)** The Pancancer IO 360 panel (NanoString Technologies) was utilized to assess the expression of 770 genes and the Tumor Inflammation Signature (TIS) using RNA that was extracted from 18 FFPE tumor samples. **(d)** From the FFPE of 25 individuals, genomic DNA (DNA that has undergone bisulfite conversion) was extracted and utilized for hybridization on an Illumina Infinium Methylation EPIC BeadChip. Figure created with BioRender.com (accessed on 23/05/2023; agreement number FD25EFE46G).

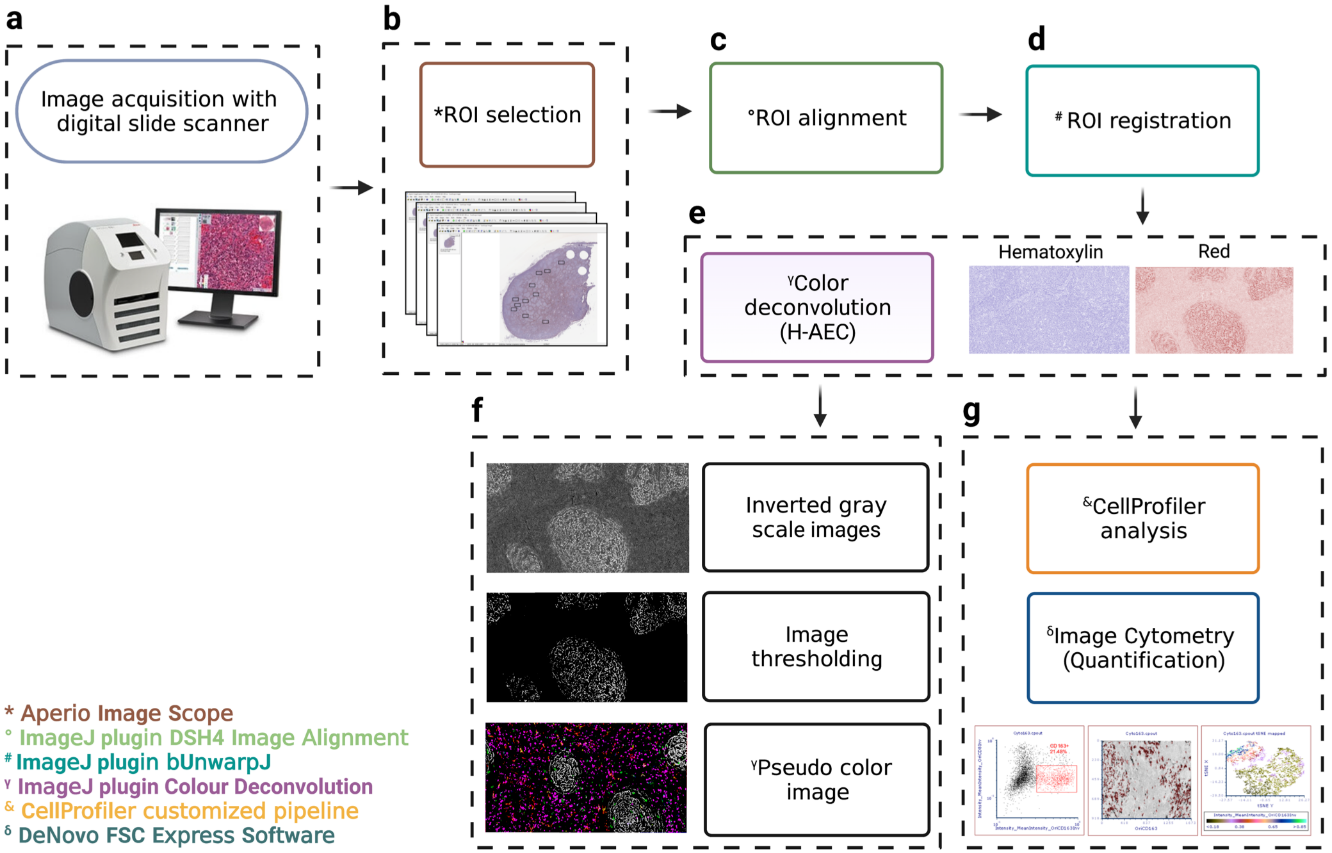

Figure S2. Flowchart of digital image processing workflow applied to sequential IHC. Computational image processing for quantification and pseudo-color image visualization was conducted using several tools: (**a**) image acquisition, performed using a commercial slide scanner Aperio CS2 (Leica Biosystems Nussloch GmbH); (**b**) ROI selection, using the commercial software *Aperio Image Scope* (Leica Biosystems Nussloch GmbH); (**c**) ROI alignment, using the open-source *ImageJ/Fiji* plugin *DS4H Image Alignment*; (**d**) ROI registration for elastic deformation, using the open-source *ImageJ/Fiji* plugin *bUnwarpJ*; (**e**) H-AEC color deconvolution, using the open-source *ImageJ/Fiji* plugin *Colour Deconvolution*; (**f**) pseudo-color image rendering and visualization, processing the images with *ImageJ/Fiji*; (**g**) image cytometry quantification, using a customized *CellProfiler* pipeline and the commercial *FCS Express* (DeNovo Software) tool. Figure created with BioRender.com (accessed on 19/01/2023; agreement number CK24WPDQ6C).

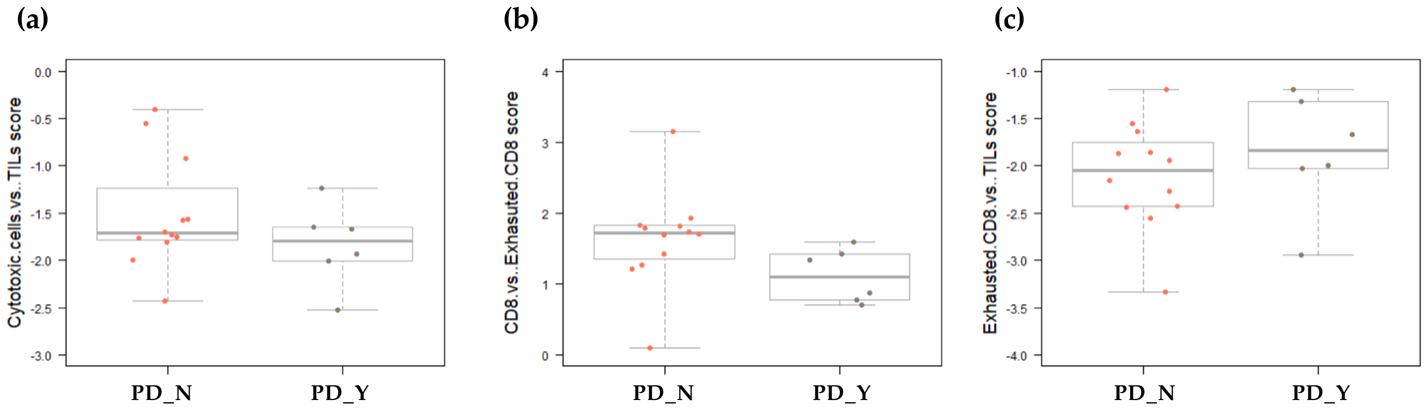

Figure S3. Nanostring BoxPlots of CD8 functionality. Box plot histograms representing the score values in DR_N and DR_Y samples analyzed using the Pancancer IO 360 Nanostring panel. **(a)** Ratio between Cytotoxic cells vs TILs gene categories. **(b)** Ratio between CD8 vs Exhausted CD8 gene categories. **(c)** Ratio between Exhausted CD8 vs TILs gene categories. Gene counts were normalized to Housekeeping and log2-transformed.

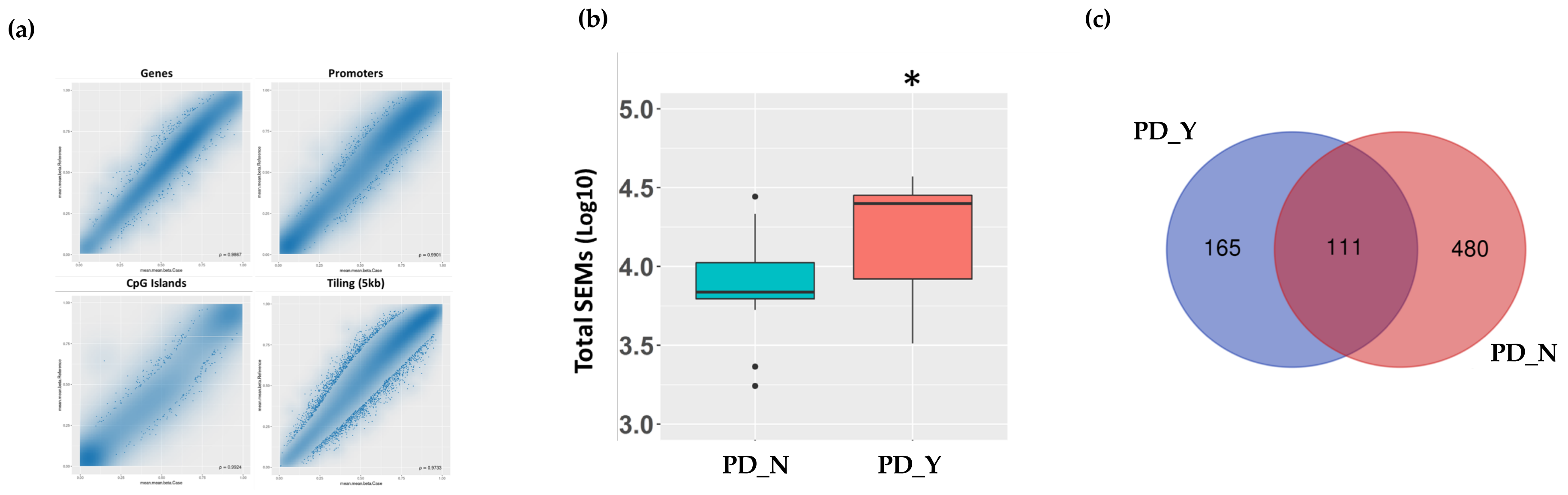

Figure S4. Stochastic Epimutations was evaluated by comparing sample groups according to Disease Recurrence. (a) Scatterplot for differential methylation (regions). By using a p-value (FDR) criterion, the transparency corresponds to point density. If the number of points exceeds 2e+06 then the number of points for density estimation is reduced to that number by random sampling. **(b)** Boxplot showing the distribution of SEMs in sample's groups. The thick horizontal line represents the median of the distribution while the box represents the interquartile range. Whiskers are set as the default option for the boxplot function and extend to the most extreme data point, which is no more than 1.5 times the interquartile range from the box. Open circles represent outliers (single values exceeding 1.5 interquartile ranges). **(c)** Venn diagram of genes resulting from PD_Y (n=276) and PD_N (n=591) population, showing univocal and shared gene lists. P-value: *<0.05

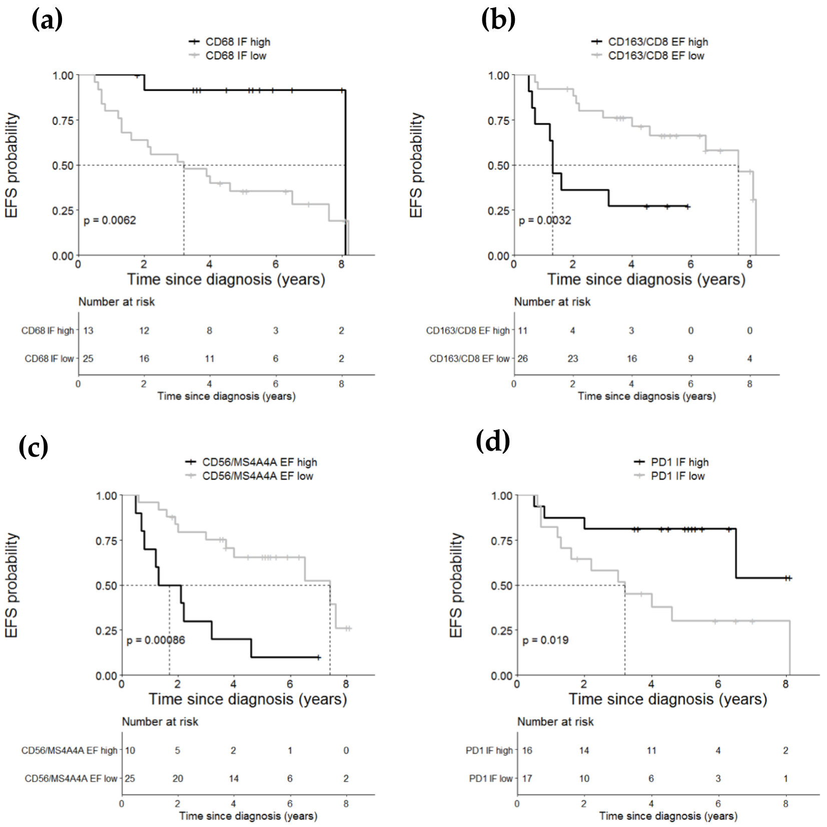

Figure S5. **Kaplan Meier analysis of EFS among treated patients with follicular lymphoma.** (a) EFS according to IF CD68+ stratified as high (n=13) and low (n=25) using the cut-off determined by recursive partitioning (31.5/HPF). (b)EFS according to CD163/CD8 EF ratio stratified as high (n=11) and low (n=26) using the cut-off determined by recursive partitioning (17.5/HPF).(c) High CD56/MS4A4A EF ratio (≥ 18/HPF ; n=10) showed a worse EFS than those with lower values (n=25). (d) PD1 IF lower (< 53.75/HPF; n=17) values were associated with better EFS than those with higher values (n=16).

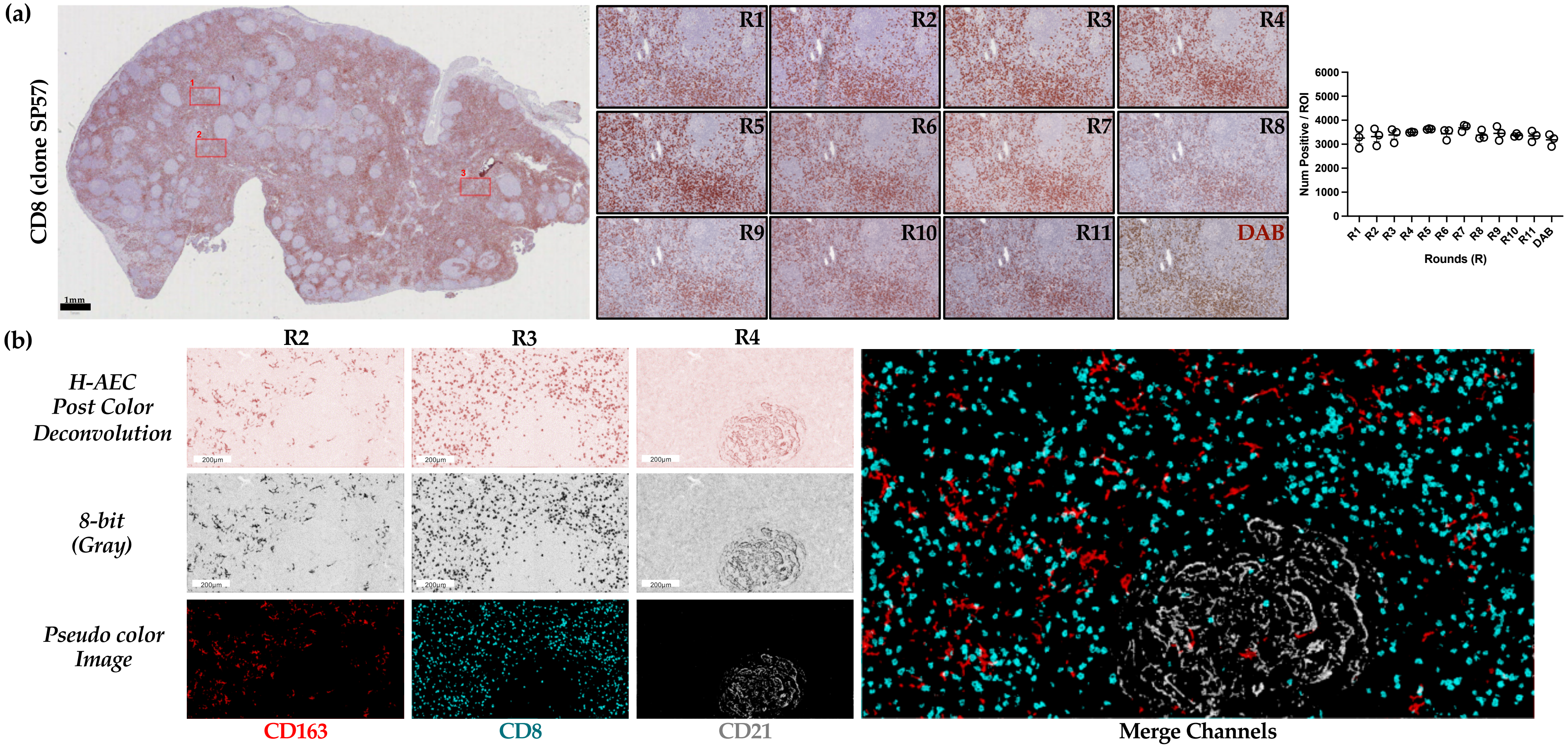

Figure S6. **Validation of the sequential IHC workflow. (a)** Illustration of the AEC staining and CD8 antibody-stripping procedure. The control tissue (human tonsil) utilized for antibody validation is shown in the left image as the WSI. The comparison between the standard IHC (DAB chromogenic staining) and the multiple rounds (R) of sequential IHC (R1 to R11) for a representative ROI is presented in the middle. The right graph displays CD8+ cell counts that were calculated using open source software QuPath for digital pathology image analysis in three Regions Of Interest (ROIs). When comparing IHC rounds 1 to 11, there was no significant loss of detection. The bars represent mean ± SEM. The Kruskal-Wallis test is used to establish statistical significance. (b) Single channel pictures of Panel A for CD163, CD8, and CD21 (R2, R3, and R4, respectively). Red images acquired after color-deconvolution are shown in the top panels, followed by 8-bit grayscale conversion in the middle, and pseudo-coloring for each individual marker in the bottom panels. The merging composite of the three markers is shown in the right image.
