# Supplementary material for: Follicular Lymphoma Microenvironment Traits Associated with Event-Free Survival
Source: Int J Mol Sci. 2023 Jun 8;24(12):9909. doi: 10.3390/ijms24129909 (PMC10297948; doi:10.3390/ijms24129909)
Supplement: Supplementary file 1 [file ijms-24-09909-s001.zip › Supplementary Tables_Tumedei et al_REVISED.pdf]

Supplementary Tables

Table S1: Detailed Clinical Pathological Characteristics of the study population.

ID	Age at diagnosis	Gender	Stage	FLIPI	Grade	BCL2
ID1	75	0	IV	I	IIIA	p
ID2#	60	0	IV	I	I	p
ID3	54	1	IV	I	II	p
ID4#	41	0	III	I	IIIA	p
ID5#	80	1	II	L	IIIA	n
ID6	46	1	IV	I	II	p
ID7#	57	1	III	L	II	p
ID8#	56	1	II	L	IIIA	n
ID9	41	1	I	L	IIIA	n/p
ID10#	63	0	III	I	I/II	p
ID12	49	1	IV	I	I/II	p/n
ID13#	42	0	II	L	IIIA	n
ID14#	87	1	III	I	IIIA	p
ID15#	37	0	II	L	IIIA	n
ID16	70	1	II	I	IIIA	n
ID17	71	1	I	L	II	p
ID18	34	0	IV	I	IIIA	p
ID19#	65	0	III	H	IIIA	n
ID20#	61	1	I	L	IIIA	n
ID21#	67	1	IV	I	II	p
ID23#	42	0	I	L	IIIA	n
ID24	38	1	I	L	IIIA	n
ID25	71	1	IV	H	IIIA	p
ID26	71	0	III	I	IIIA	p

ID27#	66	1	I	I	I/II	p/n
ID28	66	1	I	I	I	p
ID29#	65	0	IV	L	II	p
ID30	67	1	IV	H	IIIA	p
ID31#	56	1	II	I	II	n/p
ID32#	52	0	IV	H	I	p
ID33#	40	0	II	L	I/II	p
ID34#	65	0	IV	I	IIIA	p
ID35#	38	0	IV	I	IIIA	p
ID36#	61	0	II	I	IIIA	p
ID37#	63	0	I	L	II	p
ID38#	53	0	II	L	II	p
ID39#	53	1	I	L	II	n
ID40#	51	1	I	L	II	n
ID41	59	0	IV	L	II	p
ID42	55	0	II	L	IIIA	p
ID43	56	0	IV	I	IIIA	p
ID44	64	1	IV	I	IIIA	p
ID45	NA	0	IV	I	IIIA	p
ID46	52	0	II	L	I/II	p
ID47	56	0	III	H	I	p
ID48	70	1	IV	H	I	p
ID49	41	0	III	L	II	p/n
ID50	68	0	III	I	IIIA	p
ID51	58	1	IV	L	II	p

ID, Identification Code; Male, 0; Female, 1; #, samples included in the methylation analysis; FLIPI, Follicular Lymphoma International Prognostic Index; I, Intermediate; L, Low; H, High; BCL2; p, positive (including p/n); n, negative (including n/p); NA , not available.

Table S2. Staining conditions for standard and seqIHC

Antibody	Clone	Sources	Dilution (IHC; SeqIHC)	Incubation
anti-CD68	PG-M1	Dako	1:100 ; 1:25	32 minutes
anti-CD163	MRQ-26	Ventana (Roche)	ready to use	32 minutes
anti-CD8	SP57	Ventana (Roche)	ready to use	1 hour
anti-MS4A4A	Polyclonal	Atlas Antibodies	1:350	32 minutes
anti-CD56	MRQ-42	Ventana (Roche)	ready to use	32 minutes
anti-CD21	EP3093	Ventana (Roche)	NA ; Ready to use	1 hours
anti-PD1	NAT105	Ventana (Roche)	ready to use	36 minutes

Dako (Glostrup, Denmark); Roche Ventana Medical Systems (Tucson, AZ, USA); Atlas Antibodies AB (Bromma,Sweden); seqIHC, sequential IHC; NA, not applied.

Table S3: IHC Score Median Value.

ID	Median CD68 IF	Median CD68 EF	Median CD163/CD8 EF	Median CD56/MS4A4A EF	Median PD1 IF	Median PD1 EF
ID1	22,5	42,5	8	9	NA	NA
ID2#	13	36,5	10	15	45	19,5
ID3	15,5	27	NA	NA	NA	NA
ID4#	28,5	35,5	1	10	76	34,5
ID5#	18	49	35	10	76	27
ID6	32	27,5	12,5	NA	39,5	24

ID7#	12	28	9	33	36	26,5
ID8#	48,5	81,5	10	12	75	48
ID9	32	37,5	32,5	1	57	14
ID10#	29,5	29	9	4	80,5	32,5
ID12	27	40	9	5	54	44,5
ID13#	54,5	59,5	0,5	NA	51	38,5
ID14#	14	25,5	20	10	39	15,5
ID15#	34,5	67,5	6,5	10	69	46,5
ID16	23	29,5	55	NA	30,5	56,5
ID17	29	36	5	15	41,5	23
ID18	26	39,5	30	12,5	45,5	46,5
ID19#	67,5	87,5	5	2	75,5	33,5
ID20#	39	45	7,5	6,5	46	29,5
ID21#	17,5	25	2	17,5	58,5	33,5
ID23#	21	63,5	23,5	14	29	65,5
ID24	12	57,5	5	NA	41	23
ID25	21	28	57,5	45	44,5	49,5
ID26	29	27	9	27,5	NA	NA
ID27#	32	103,5	10	10	57,5	34,5
ID28	19,5	22,5	10	NA	NA	NA
ID29#	33	47	7,5	7,5	94	27
ID30	24,5	56,5	10	42	77,5	36
ID31#	36	62,5	37,5	5	37	29,5
ID32#	28	97	10	4	25	11,5
ID33#	27	31	2	15	77	48
ID34#	16,5	44,5	3	NA	94,5	44,5
ID35#	20	46	22,5	40	68	34,5

ID36#	19,5	54,5	12,5	20	40	45
ID37#	41,5	61,5	5	12,5	44	29
ID38#	39,5	50,5	7,5	1	78	91,5
ID39#	37,5	68	22,5	15	59.5	30,5
ID40#	44	64	9,5	2,5	78,5	31
ID41	16	32,5	40	9	48	27,5
ID42	18	43	9	4,5	47	24
ID43	22,5	50,5	5	35	79,5	34,5
ID44	27	38	50	3,5	53,5	47
ID45	13,5	27	6	18,5	51,5	67,5
ID46	14,5	78,5	24	18,5	NA	NA
ID47	12,5	36	60	8	46	38,5
ID48	17	64,5	4,5	5	37	19
ID49	31	22	28	22	34	35,5
ID50	25	33	25	NA	38,5	30,5
ID51	17	67,5	3,5	34	40,5	28,5

ID, Identification Code; #, samples included in the methylation analysis; NA, not available; IF,intrafollicular; EF extrafollicular.

Table S4: Sequential IHC Manual Protocol Summary for Panel A.

	**Round 1**	**Round 2**	**Round 3**	**Round 4**
Deparaffinization				
Xilene	40 minutes	NA	NA	NA
Rehydration				
100% ETOH	2 minutes	NA	NA	NA

100% ETOH	2 minutes	NA	NA	NA
90% ETOH	5 minutes	NA	NA	NA
70% ETOH	5 minutes	NA	NA	NA
50% ETOH	5 minutes	NA	NA	NA
H20 DEMI	2 minutes	NA	NA	NA
Antigen Retrival	Ultra CC1 (Roche)	Ultra CC1 (Roche)	Ultra CC1 (Roche)	Ultra CC1 (Roche)
Temperature	100°C	100°C	100°C	100°C
Time	60 minutes	40 minutes	40 minutes	40 minutes
Protein Blocking	PBS +1% BSA	PBS +1% BSA	PBS +1% BSA	PBS +1% BSA
Temperature	RT	RT	RT	RT
Time	20 minutes	20 minutes	20 minutes	20 minutes
H2O2 Blocking	15 minutes	15 minutes	15 minutes	15 minutes
Primary Antibody	CD68 (PG-M1 Dako)	CD163 (MRQ-26 Roche)	CD8 (SP57 Roche)	CD21 (EP3093 Roche)
Diluition	1:25	Ready to use	Ready to use	Ready to use
Temperature	RT	RT	RT	RT
Time	1 hours	1 hours	1 hours	1 hours
Secondary Antibody	Reagent A (Genemed)	Reagent A (Genemed)	Reagent A (Genemed)	Reagent A (Genemed)

Diluition	Ready to use	Ready to use	Ready to use	Ready to use
Temperature	RT	RT	RT	RT
Time	30 minutes	30 minutes	30 minutes	15 minutes
AEC Chromogen (Genemed)	40 minutes	35 minutes	30 minutes	30 minutes
Counterstain	Hematoxylin	Hematoxylin	Hematoxylin	Hematoxylin
Stripping				
Temperature	90°C	90°C	90°C	90°C
Time	20 minutes	20 minutes	20 minutes	20 minutes

Dako (Glostrup, Denmark); Roche Ventana Medical Systems (Tucson, AZ, USA); Genemed Biotechnologies (Torrance, CA 90501, USA).

Table S5: : Sequential IHC Manual Protocol Summary for Panel B.

	Round 1	Round 2	Round 3
Deparaffinization			
Xilene	40 minutes	NA	NA
Rehydration			
100% ETOH	2 minutes	NA	NA
100% ETOH	2 minutes	NA	NA

90% ETOH	5 minutes	NA	NA
70% ETOH	5 minutes	NA	NA
50% ETOH	5 minutes	NA	NA
H20 DEMI	2 minutes	NA	NA
Antigen Retrival	Ultra CC1 (Roche)	Ultra CC1 (Roche)	Ultra CC1 (Roche)
Temperature	100°C	100°C	100°C
Time	40 minutes	40 minutes	40 minutes
Protein Blocking	PBS +1% BSA	PBS +1% BSA	PBS +1% BSA
Temperature	RT	RT	RT
Time	20 minutes	20 minutes	20 minutes
H2O2 Blocking	15 minutes	15 minutes	15 minutes
Primary Antibody	CD163 (MRQ-26 Roche)	MS4A4A (Atlas Antibodies)	CD21 (EP3093 Roche)
Diluition	Ready to use	1:350	Ready to use
Temperature	RT	RT	RT
Time	1 hours	1 hours	1 hours
Secondary Antibody	Reagent A (Genemed)	Reagent A (Genemed)	Reagent A (Genemed)
Diluition	Ready to use	Ready to use	Ready to use

Temperature	RT	RT	RT
Time	30 minutes	30 minutes	15 minutes
AEC Chromogen (Genemed)	35 minutes	30 minutes	30 minutes
Counterstain	Hematoxylin	Hematoxylin	Hematoxylin
Stripping			
Temperature	90°C	90°C	90°C
Time	20 minutes	20 minutes	20 minutes

Roche Ventana Medical Systems (Tucson, AZ, USA); Atlas Antibodies AB (Bromma, Sweden); Genemed Biotechnologies (Torrance, CA 90501, USA).

Table S6: : Sequential IHC Manual Protocol Summary for Panel C.

	**Round 1**	**Round 2**	**Round 3**	**Round 4**
Deparaffinizatio n				
Xilene	40 minutes	NA	NA	NA
Rehydratation				
100% ETOH	2 minutes	NA	NA	NA
100% ETOH	2 minutes	NA	NA	NA
90% ETOH	5 minutes	NA	NA	NA

70% ETOH	5 minutes	NA	NA	NA
50% ETOH	5 minutes	NA	NA	NA
H20 DEMI	2 minutes	NA	NA	NA
Antigen Retrival	Ultra CC1 (Roche)	Ultra CC1 (Roche)	Ultra CC1 (Roche)	Ultra CC1 (Roche)
Temperature	100°C	100°C	100°C	100°C
Time	60 minutes	40 minutes	40 minutes	40 minutes
Protein Blocking	PBS +1% BSA	PBS +1% BSA	PBS +1% BSA	PBS +1% BSA
Temperature	RT	RT	RT	RT
Time	20 minutes	20 minutes	20 minutes	20 minutes
H2O2 Blocking	15 minutes	15 minutes	15 minutes	15 minutes
Primary Antibody (Clone, Supplier)	PD1 (D4W2J, Cell Signaling)	CD163 (MRQ-26, Roche)	CD8 (SP57, Roche)	CD4 (EPR6855, Abcam)
Diluition	1:50	Ready to use	Ready to use	1:100
Temperature	RT	RT	RT	RT
Time	1 hours	1 hours	1 hours	1 hours
Secondary Antibody	Reagent A (Genemed)	Reagent A (Genemed)	Reagent A (Genemed)	Reagent A (Genemed)
Diluition	Ready to use	Ready to use	Ready to use	Ready to use

Temperature	RT	RT	RT	RT
Time	30 minutes	30 minutes	30 minutes	15 minutes
AEC Chromogen (Genemed)	30 minutes	35 minutes	30 minutes	30 minutes
Counterstain	Hematoxylin	Hematoxylin	Hematoxylin	Hematoxylin
Stripping				
Temperature	90°C	90°C	90°C	90°C
Time	20 minutes	20 minutes	20 minutes	20 minutes

Cell Signaling (Danvers, Massachusetts, USA); Roche Ventana Medical Systems (Tucson, AZ, USA) ; Abcam (Cambridge, UK); Genemed Biotechnologies (Torrance, CA 90501, USA).

Table S7. Differentially expressed genes emerged from the Nanostring pancancer IO 360 panel.

Probe Name	true	StDev of true	false	true vs. false	P value of: true vs.false	t-statistic of: true vs. false
HLA-DQA1	8536,77	2762,15	265,91	32,1	0,01121703	3,024616
HLA-DQB1	2388,64	1451,38	102,2	23,37	0,00600739	3,32830429

PRLR	5,36	4,61	1,56	3,43	0,00481089	3,58396196
EXO1	111,3	77,74	51,64	2,16	0,03992193	2,24308014
HLA-DRB1	15117,88	8082,87	7054,71	2,14	0,02227121	2,67101026
FANCA	505,24	266,66	260,57	1,94	0,04875314	2,14378262
HDAC11	37,22	22,94	19,51	1,91	0,04117578	2,38626385
NEIL1	565,1	213,84	303,04	1,86	0,02629859	2,44772577
CDC20	130,81	36,41	78,57	1,66	0,02773041	2,42123103
ICOSLG	548,1	215,95	351,07	1,56	0,04840287	2,15330195
SELP	46,68	16,07	69,1	-1,48	0,049953	-2,24219918
LYZ	6826,87	1715,33	10807,65	-1,58	0,02138085	-2,55125451
STAT4	170,55	51,85	281,39	-1,65	0,02253814	-2,56660795

FBP1	41,96	24,12	77,29	-1,84	0,04288103	-2,28705525
GZMA	124,69	68,9	239	-1,92	0,03750603	-2,37910557
COL6A3	203,56	36,09	428,46	-2,1	0,00888636	-3,07012916
CXCR3	34,8	13,26	74,22	-2,13	0,00309037	-3,51935434
CCR2	34,97	28,65	74,97	-2,14	0,04085206	-2,42995501
TRAT1	97,13	55,93	209,69	-2,16	0,01732074	-2,92710161

Table S8. Cumulative survival table.

ID	Age	T0	Date of Treatment Initiation	Treatment Response	PD	PDdate	CD163/CD8 EF	CD68 IF	CD68 EF	PD1 IF	PD1 EF	CD56/MS4A4A EF	POD24	Time to PD

ID1	75	13/9/2004	22/10/2004	CR	1	8/5/2012	8	22,5	42,5	NA	NA	9	N	7,6
ID2#	60	29/3/2006	14/6/2006	CR	1	2/4/2010	10	13	36,5	45	19,5	15	N	4
ID3	54	5/5/2006	3/7/2006	CR	1	26/3/2010	NA	15,5	27	NA	NA	NA	N	3,9
ID4#	41	29/9/2006	4/5/2011	CR	1	13/4/2013	1	18	35,5	76	34,5	10	Y	7,2
ID5#	80	28/3/2008	NA	NA	1	NA	35	18	49	76	27	10	Y	NA
ID6	46	31/8/2009	15/10/2009	CR	1	22/9/2017	12,5	32	27,5	39,5	24	NA	N	8,1
ID7#	57	6/11/2009	20/1/2010	CR	1	2/1/2012	9	12	28	36	26,5	33	Y	2,2
ID8#	56	20/1/2010	15/4/2010	CR	0	21/5/2015	10	48,5	81,5	75	48	12	N	5,3
ID9	41	21/1/2010	4/3/2010	CR	0	2/4/2015	32,5	32	37,5	57	14	1	N	5,2

ID10#	63	11/3/2010	29/3/2010	CR	0	28/4/2018	9	29,5	29	80,5	32,5	4	N	8,1
ID12	49	16/4/2010	17/5/2010	CR	0	14/5/2015	9	27	40	54	44,5	5	N	5,1
ID13#	42	4/5/2010	3/6/2010	CR	0	7/11/2016	0,5	54,5	59,5	51	38,5	NA	N	6,5
ID14#	87	25/2/2010	NA	CR	1	13/11/2013	20	14	25,5	39	15,5	10	N	3,7
ID15#	37	16/7/2010	9/9/2010	CR	0	17/1/2014	6,5	34,5	67,5	69	46,5	10	N	3,5
ID16	70	13/8/2010	NA	PD	1	1/10/2010	55	23	29,5	30,5	56,5	NA	Y	0,1
ID17	71	27/8/2010	NA	NA	1	21/1/2018	5	29	36	41,5	23	15	N	7,4
ID18	34	25/3/2011	4/5/2011	PR	1	9/11/2011	30	26	39,5	45,5	46,5	12,5	Y	0,6
ID19#	65	6/9/2011	1/3/2012	CR	0	20/9/2019	5	67,5	87,5	75,5	33,5	2	N	8

ID20#	61	5/9/2012	30/1/2012	CR	0	27/5/2016	7,5	39	45	46	29,5	6,5	N	3,7
ID21#	67	10/10/2011	15/11/2011	CR	0	8/2/2018	2	17,5	25	58,5	33,5	17,5	N	6,3
ID23#	42	NA	24/1/2010	CR	0	NA	23,5	21	63,5	29	65,5	14	N	NA
ID24	38	3/2/2012	NA	NA	0	27/2/2018	5	12	57,5	41	23	NA	N	6,1
ID25	71	22/3/2012	18/4/2012	CR	1	20/5/2015	57,5	21	28	44,5	49,5	45	N	3,2
ID26	71	18/4/2012	26/6/2012	CR	1	19/5/2014	9	29	27	NA	NA	27,5	Y	2,1
ID27#	66	6/6/2012	6/6/2012	CR	1	17/4/2014	10	32	103,5	57,5	34,5	10	Y	1,9
ID28	66	14/6/2012	4/7/2012	CR	1	10/8/2020	10	19,5	22,5	NA	NA	NA	N	8,2
ID29#	65	15/11/2012	10/1/2013	PR	0	1/7/2016	7,5	33	47	94	27	7,5	N	3,6

ID30	67	7/12/2012	1/2/2013	PD	1	1/10/2013	10	24,5	56,5	77,5	36	42	Y	0,8
ID31#	56	27/2/2013	12/3/2013	CR	0	6/2/2019	37,5	36	62,5	37	29,5	5	N	5,9
ID32#	52	NA	27/8/2010	CR	1	NA	10	28	97	25	11,5	4	Y	NA
ID33#	40	27/3/2013	15/5/2013	CR	0	12/3/2018	2	27	31	77	48	15	N	5
ID34#	65	15/5/2013	23/7/2013	PR	0	13/9/2017	3	16,5	44,5	94,5	44,5	NA	N	4,3
ID35#	38	18/7/2013	25/7/2013	PR	1	20/1/2014	22,5	20	46	68	34,5	40	Y	0,5
ID36#	61	30/7/2013	23/9/2013	CR	0	10/8/2020	12,5	19,5	54,5	40	45	20	N	7
ID37#	63	13/8/2013	7/10/2013	CR	0	20/5/2015	5	41,5	61,5	44	29	12,5	N	1,8
ID38#	53	26/8/2013	1/10/2013	CR	0	8/3/2019	7,5	39,5	50,5	78	91,5	1	N	5,5

ID39#	53	23/10/2013	23/1/2014	CR	0	7/5/2018	22,5	37,5	68	59,5	30,5	15	N	4,5
ID40#	51	24/12/2013	24/10/2014	CR	1	10/1/2016	9,5	44	64	78,5	31	2,5	Y	2
ID41	59	12/11/2014	NA	CR	NA	NA	40	16	32,5	48	27,5	9	N	NA
ID42	55	13/3/2015	13/3/2015	CR	NA	NA	9	18	43	47	24	4,5	N	NA
ID43	56	27/3/2015	27/3/2015	CR	NA	NA	5	22,5	50,5	79,5	34,5	35	N	NA
ID44	64	8/5/2015	NA	CR	1	12/12/2016	50	27	38	53,5	47	3,5	Y	1,6
ID45	NA	14/4/2016	1/7/2016	PD	1	3/1/2017	6	13,5	27	51,5	67,5	18,5	Y	0,7
ID46	52	3/6/2016	15/5/2016	CR	1	12/9/2017	24	14,5	78,5	NA	NA	18,5	Y	1,3
ID47	56	1/4/2016	10/6/2016	CR	1	12/7/2017	60	12,5	36	46	38,5	8	Y	1,3

ID48	70	1/1/2017	13/3/2018	CR	1	8/1/2020	4,5	17	64,5	37	19	5	Y	3
ID49	41	22/2/2018	12/5/2018	PR	1	16/5/2019	28	31	22	34	35,5	22	Y	1,2
ID50	68	18/7/2019	26/7/2019	PD	1	1/4/2020	25	25	33	38,5	30,5	NA	Y	0,7
ID51	58	15/9/2013	24/10/2013	CR	1	13/4/2018	3,5	17	67,5	40,5	28,5	34	N	4,6

ID, Identification Code; #, Methylation Analysis; T0, Time of Diagnosis; CR, Complete Response; PD, Progression Disease; PR, Partial Response; NA , not available; 0, PD_N; 1, PD_Y; EF, extrafollicular; IF, intrafollicular;

Table S9. SEM-enriched genes according to progressive disease.

SEM-enriched genes in the PD positive group (n= 165)	*LYPD6B, ORC3, PXT1, MYO9B, IQCK, SERGEF, CLGN, PRKCZ, GUSBP5, TTC31, CDC14B, RAB3C, NFIA, HHEX, PCYT2, HES5, EPS8, STX17, BMPR2, PAPSS2, PFKFB2, CPSF1, SH3BP2, GPC5, TENM4, GSX2, EPB41L2, MEF2C-AS2, LOC100130691, LOC101928063, MFAP3, SOCS6, KCNA3, DHX38, FABP5, NOP9, PDGFA, MFSD11, PAFAH2, LINC01635, NABP2, ULK1, NFKB2, SCP2, BLCAP, SLC25A27, DUSP5, TBC1D17, SLCO3A1, VIM-AS1, DHDDS, FBXO24, PIGV, TRAF3, DPYD, PTGES3, TM2D2, LOC101928386, SAMD12-AS1, ARL4C, BBS1, ISOC2, VASH1, HIPK1-AS1, ULBP3, KCNQ5, AGPS, TAPBP, PKIB, TCF4, TTC39A-AS1, ALDH1L2, STX17-AS1, OTX1, NTNG1, NABP1, MIR4638, PRRC2A, DGCR6, TMUB1, KCNC4, HCK, SRSF2, ACVR2B, RNPS1, ZNF146, TPT1, SOX9, CDC42SE1, TGIF1,, RNH1, BEND6, LOC105375401, PRPSAP2, ALS2CL, GACAT2, EFNA1, CHURC1-FNTB, VIM, WARS,, MRPS21, PATJ, LOC153684, B4GALT6, ZNF518B, SYDE2, GABRB2, CCND2, FHIT, ADCY4, KANK3, BEND7, CHURC1, FDFT1, PITX3, DDAH2, BCAR1, UCHL5, GAMT, PREX1,CELSR3-AS1, GK5, PPP1R14C, LOC101927752, TOX, F12, LOC648987, TXNL4B ,GTF2H4, ZNF565,DHRS1,MIR5091,CCDC179,CD9,LOC105378591, LRIG1, RIMS1, PMP22, AMIGO3, HPGD, GDF7, DCUN1D2, VARS2, OCIAD1, TFPI2, TRNP1, RNF123, SOX9-AS1, CPQ, ACVR2B-AS1, MAVS, NOTCH1, AGAP2, SOX18, CEP104, HLA-F-AS1, AGAP2-AS1, DISP3, ZMYM1, ZNF215, PRR3, C3orf67, ANKZF1, PDIA5, KCTD20*
SEM-enriched genes in the PD negative group (n=480)	*FERMT2, TBC1D19, HACD1,GSG1L,SMARCD3, LOC102724360, KIF26B, KIFC3, LOC728485, RTKN2, HSPB1, TIGD7, PFKP, COL9A3, MFSD2B, PRKCSH, DOC2A, HLA-F, ZSCAN18, FAN1, RGMB, PAM, HNRNPH1, MAPT-IT1, PACRG, ABCG2, FRAS1, LOC101927989, RHPN1, MMP28, GNAL, PRKN, EPHA3, RTN1, P4HA2, LOC283856, FNBP1L, SEMA5B, RNF220, ZNF337-AS1, FAM151B, G2E3, TSPAN15, TFDP2, TYW3, CCDC28B, MSRB3, NETO2, ZDHHC1, FAM200A, MET, RNU5F-1,PTPRE, SYNPO2, CRABP2, RAB40B, NR4A2, PTPDC1, LINC01305, FBXL15, ZNF582-AS1, KCNQ4, HOXA13,FIBCD1, ZNF718, B4GALNT4, CACNB4, ITGA2B, CEND1, JAM3, SLC39A7, NTRK3-AS1, B4GALNT1, NXPH3, ZNF582, IFT172, NTRK2, LOC101928222, POC1B-AS1, PXDC1, AGRN, LOC105370333, LOXL2, ABCG1, TMEM56-RWDD3, TSKU, LRGUK, GAREM2, B3GNT9, ZNF571, PTOV1, SCOC, ZNF354B, TBC1D12, PSD, SPRY1, TRIM4,CCDC39, ELOVL3, LTBP4, COL4A4, TTLL6, ZDHHC16, GJA3, NKD1, ZNF808, ZNF345, NAT8L, ENOX1, SNX5,RING1, LOC102724404, PTOV1-AS1, CTIF, MSRB2, CYFIP1, LOC728392, GALK2, ZNF385B, TSPO, ATP11A, SEMA6C, GLMN, ARHGAP19-SLIT1, AP4B1-AS1, ATP1B1, ZNF844, PLEKHG4, SAMD11, SDC1, SMAD1, NEUROG3, MARK1, FRMD5, SLC22A15, TFCP2L1, HSPA14, IQCJ-SCHIP1, PAK6, ZNF773, DDAH1, SLC16A5, CSF1, TUBB, COX18, WDR35, FBXL19-AS1, MGME1, ZBED9, CCNI2, CHFR, RBM11, SH3D21, PRR34, TNXB, THRB, MYEF2, KLF5, C2CD4D, RBM26-AS1, PDE5A, KHDRBS3, SLC4A3, ARAP2, SLC30A10, MAGI3, LOC101928977, CDKN2B-AS1, LOC100505938, TFR2, PURG, SNAP25-AS1, PLEKHH1, AS-PTPRE, NTNG2, SIX4, TLCD2, LOC100272217, GPR19, PTPRG, LOXL1-AS1, MOCOS, SPATA18, HLDA1, SDC2, PRR5L, HPDL, WNT3, ZNF549, CRYZ, PALM2, FGFR3, EEF1AKMT3, RSBN1, PRR34-AS1, ABCB4, SMOC1, FER, CFL1P1, LOC646548, SETDB2, FUT4, HIST1H3C, KIFC1 ,RAB26, TOGARAM1, KCNK13, COL4A3,MAP2, ZNF853, MAPT, MMP17, FADS2, CYP51A1-AS1, LINC00535, SOX2-OT, LINC00466, CBSL, TBX2-AS1, DST, LOC439933, ISPD, ZNF540, C1orf21, CCDC85C, TP53I11, STON1, TMEM200B, THCAT158, RHBDF2, ZNF606, RTN4RL1, TNFSF12, PBX1, ERICH5, ITFG1-AS1, FAM171A1, LOXL1, THOC6, NR2E1, ADORA2B, PRDX2, LOC100287944, ARHGEF16, BBS5, SCHIP1, SKAP2, GNAO1, FSTL4, GDF9, PCCA, ACCS, LTBP2, ATP7B, USP4, SLC35G2, BMP6, PPL, ZNF532, ZNF585B, VCAN, SLFN11, HDAC11-AS1, SOCS2, PCYOX1L, LEF1, ZNF816-ZNF321P, TXNRD2, LOC105376671, ICA1, CEBPD, EMID1, MAP1LC3A, POC1B-GALNT4, C1orf53, CTBP2, NDRG2, ZMYND11, C15orf56, MRPS33, GPR137, SPOCK3, LOC101926898, SLC16A9, LMCD1-AS1, CRACR2A, WWC1, MAPT-AS1, TTC23L, TGFBR3, ST8SIA1, MYCBPAP, LOC283045, TMEM229B, PDE8A, ZNF615, MYCNOS, LDLR, ZNF516, RBM26, BUB1B-PAK6, CAT, ZNF649, HIST1H2BB, PPP2R2B, EIF3D, LEF1-*

	AS1, PDGFB, LOC101930010, TCTN2, TMEM256-PLSCR3, GPD2, SPR, SCUBE2, VANGL1, ACSL6, PTK7, NRBP2, NDRG4, LOC100132111, C20orf194, MAN1C1, RAB20, ECHDC2, TET1, RHPN1-AS1, RAB39A, LOC101928464, ABCB6, MYH10, LOC101928530, PPIC, KCNQ3, HOXA-AS3, SOWAHA, TCTA, LOC101928837, LOC102724450, NKILA, HCFC1R1, SPRED1, LMCD1, CBS, SLC26A4, ENC1, RPAP2, ATRNL1, BORCS7-ASMT, TIMP2, DNMT3A, OVOL1, C1QTNF1-AS1, BAD, HEXB, ACVR1C, SALL2, LOC100128398, TUBGCP5, HHIPL1, SMPDL3A, LRRC61, HPSE, SLC35G1, ARHGEF25, HMGN3-AS1, IL15, GABARAPL1, ADSS, GPRC5B, TRPC1, TCF7, STK33, SCOC-AS1, ITM2C, SCCPDH, VIPR1, LOC100129697, MIR1258, ECHDC3, PMEPA1, ZNF703, SPATS2L, CYB5A, PRKACB, C3orf80, PDE1B, UQCRQ, CDR2L, TLR2, VEGFA, CCDC40, KIF9, LINC01397, PTCH1, CDKL2, FAM213A, MAST4, DDX46, ANXA4, ALKBH3, TES, HOXB7, PRKAG2, OSBPL6, MOCS2, RFX8, CYBRD1, SPTSSB, ZC3H12C, CAST, ZNF57, FBN1, GAB1, SLC26A4-AS1, ARHGAP10, EPHB2, STON1-GTF2A1L, PIANP, GATM, LOC339874, RASA3, TXLNA, TMEM56, SLC4A4, SETDB2-PHF11, MAGI1, FJX1, CPPED1, TOB2P1, IQCC, CUTA, STUM, AOX1, MSANTD4, ZNF816, TRIM36, NUDT16, TSPAN5, EDEM3, FAM92A, PRKCA, TTC38, PRMT7, NIM1K, RHBDL1, BAIAP2, RASSF1, TRPC3, KCNS3, ACADS, ZMYND10, EPHB1, DPYSL3, HOTTIP, CH25H, CDC7, LOC101927809, REEP1, ZNF358, ELMOD1, FCHSD1, RHBDD1, CD109, RBMS1, GRID1, RPS6KA2, TNFSF12-TNFSF13, EFR3B, RPRML, CHRNA7, LOC105379152, ELOC, ZNF304, ABHD1, CDIP1, CBFA2T3, ZNF461, ACTR3C, C3orf62, RXRB
SEM-enriched shared genes	*CACNA1C, TMEM178B, TBC1D4, MSC-AS1, MSC, PHLDA3, IGF2BP2,, SOX4, NEK5, KDELC2, FADS1, ARHGAP21, CMPK2, PYGL, SOX5, ZNF793, FAM117A, SEMA6A-AS2, BIVM, TBC1D30, LOC101928489,DUSP4, DOK1, ARL4D, POLR2F, NAXD, BDNF, PSAT1, ZNF69, ATOH8, MEIS2, ZNF75A, LINC00461, ZNF501, GLUL, ZNF30, AS1, STMN3, TBX2, F2R, HLTF-AS1, ZNF256, HCG17, ETS2, ZNF423, C11orf45, RCAN2, KDELC1, TANC1, CCND2-AS1, LZTFL1, VSIG8, LOXL3, DLG5-AS1, PROKR1, OLFML2B, COQ10B, ACVR2A, CLIP4, ZNF793-AS1, ZNF470, HLA-L, ZNF568, DGKZ, ZNF496, SLC1A2, TCF7L2, RPP25, SVIP, ENO4, AJUBA, ZFP30, FZD6, EIF5A2, LOC101929657, P2RY2, SLC27A2, PYCR1, C11orf96, ZNF570, ZNF790, SHOX2, RSPO3, ZNF30, ZNF134, ZSWIM6, ACTN1-AS1, KCNJ5, ACTN1, SNHG28, GNL1, BCAT1, DLX1, USMG5, MME, S100A6, FMNL2, PTH1R, C22orf23, THRB-AS1, GAD1, USP44, ZNF829, GALNT16, ZNF74, GPAT3, HSPA12A, SEMA6A, WFS1, CACNA2D3, KCTD1, POLR3B*
